# Intelligence

**Published:** 1808-11

**Authors:** 


### INTELLIGENCE.

ACCOUNTS have been received from various parts of the flat countries, concerning the unusual prevalence of ague during the last autumn. The disease is said to have been attended with more than common severity, with several anomalous or irregular symptoms, and to have yielded with more difficulty to the general remedies. The Editors have also been informed, that in some of the low grounds which have been drained, fevers have prevailed of a low type, which though rarely fatal, have been extremely tedious. On any of these subjects they will be thankful to be informed by the gentlemen practising in those districts. They particularly request that such as are too much engaged to arrange their notes in a manner they would wish to meet the public eye, will favour them with as many facts as they can satisfactorily authenticate, which shall be acknowledged in any manner most agreeable to the writers.

Such

Such as have more leisure, will have the goodness to be as particular as possible on the situation of the country, which is the sphere of their practice, on the usually prevailing diseases, on any changes in the same, which within their memory may have taken place, on any alteration in the cultivation of the soil, on any epizootic diseases, and if any analogy can be discovered between them and epidemics.

---

On the 7th of this month, a man was taken into Guy's Hospital, under the care of Mr. COOPER, for Popliteal Aneurism; and on the same day, about one o'clock, he was brought into the theatre of that hospital to undergo the operation of tying the femoral artery. Mr. Cooper had scarcely divided the integuments, when the man fainted, and soon after fetching three or four deep inspirations (which were supposed a prelude to his recovery) he expired. Upon the succeeding day the body was opened in the presence of the pupils. Upon puncturing tbe pericardium, it was found full of coagulated blood, which being weighed, amounted to 16 ounces troy; upon further examination it appeared, that an aneurism of considerable size had been formed in the aorta, just after its origin from the heart. This aneurism had given way during the operation, and of course produced the sudden death of the patient.

The Honourable the Governor of Madras, in council, has resolved that a reward of 6000 star pagodas, or 2000l. shall be paid to any commander of a British vessel, who may import alive at Madras the genuine cochineal insect, the growth of South America. The following description of the species of insect, for which this reward will be paid, and of the mode recommended to be pursued for the accomplishment of this object, is published for general information. There is a distinction in trade of four kinds, viz. Mestique, Compreschane, Tetruschaie, and Sylvester, of which the first is accounted the best and the last, the worst The three first derive their names from the situation; the last is found wild, and though perhaps superior to the spurious insect procured in the East Indies, is not considered as a desideratum. If either of the other three kinds above specified could be procured, it is suggested that the live insect may be preserved on the plant, during the voyage to Madras; but as the success of this experiment on a sea voyage must be precarious, every other practicable mode that could be devised, should be attempted for the purpose. The following is understood to be the mode practised by the Spaniards, for preserving the insect, while propagating its species, or depositing its eggs. The insects destined for this purpose, are taken at a proper time of the growth, put into a box well closed, and lined with coarse cloth; in this confinement they deposit their eggs and die. The box is kept close shut till the time of placing the eggs on the nopal. The animalculæ are so minute, as to be scarcely perceptible. They are put on the tree in May or June, and in two months attain to the size of a dog-tick.

The

The mode of preserving the insect on the plant, should however also be attempted, especially as there is reason to doubt, whether that on which the Spanish coccus feeds, be the same with the nopal andersoniana.

Mr. George Atkins has published a description of an improved hydrometer of his contrivance. It consists of a bulb, a small stem, with a cup on its top to receive weights, and a shank beneath the bulb with a pointed screw, to which is affixed a cup to receive weights or solids, when their specific gravities are to be taken. The instrument is accompanied with an accurate set of grain-weights. The weight of the hydrometer itself is seven hundred grains, and on adding three hundred grains in the upper cup, and immersing it in distilled water, at the temperature of 60° Fahr. it will subside to the middle mark on the stem, and will then consequeutly displace one thousand grains of water. It follows therefore, from this adjustment of the bulk of the instrument, that each grain in the upper cup will represent one-thousandth part of the specific gravity of the water, or one unit in specific gravity, if that of water be taken at one thousand; and one-tenth of a grain one-tenth of unit, which is also the value of each of the small divisions on the stem; and accordingly when the hydrometer is immersed in any liquid until it sinks to the middle point on the stem, the specific gravity of such fluid will be indicated by the sum of the weight of the instrument, and the grains added in the upper cup. To accommodate it to the use of those who are concerned with spirituous liquours and of brewers, the inventor attaches a scale, shewing the relation between specific gravities, and the commercial or technical denomination of per centage with the former, and pounds per barrel with the latter.

A Correspondent, in a letter to the Editors, writes as follows: "The rapidly increasing occurrence of fatal hydrophobia, has justly engaged the attention of many of your correspondents. As medical science is best promoted by an accumulation of well authenticated facts, perhaps you will not deem the following, which I have carefully investigated, unworthy of some mention in your articles of intelligence. — A girl, about three years and a quarter old, was standing on the upper steps of a stair case which descended into a cellar from a court in Rose-street, Soho, her face elevated about a foot above the pavement of the court. A small eur dog ran down the court, and on his return bit the child in a furious manner over both eyes, and, it was believed, would have torn her face in a dreadful manner, if her cries had not brought the neighbours to her assistance. A surgeon was immediately sent for, who treated the wounds in the usual way, not suspecting them to have been inflicted by a rabid animal. In *four weeks* from that time, within a day, about 7 P. M. on a Saturday, the symptoms of violent erethism commenced, and on Monday, at 7 P. M. she died. An invincible repugnance to swallowing any thing, either solid or fluid, continued during the whole 48 hours. Several physicians sicians and surgeons saw this case on the Monday, when it was plain that the time of preventing or curing the disease had passed over."

M. V. Aubie, apothecary of Valence, has recently made a number of chemical experiments, on the saccharine matter contained in the stalk of Indian corn. The results deduced from this examination are: 1. That the stalk of Indian corn cannot be employed for the extraction of sugar, because the expence would exceed the profit; since one hundred weight yields only two pounds of saccharine matter. 2. That this saccharine matter constantly retains the consistence of treacle, and is incapable of being crystalized by any known process. 3. That the gummy extract might be employed in medicine, as an attenuant, in consequence of its saponaceous quality.

A capuchin of Vicenza, named John Baptist de Saint Martin, has invented a very useful instrument for ascertaining the quantity of saccharine matter in unfermented wine, and showing how to extract it. This instrument, called an *enometer*, has been examined by the academy of Sciences at Naples, who were satisfied that it fully answered the purposes for which it was intended.

Some time in January, Dr. Reid will resume his Lectures on the Theory and Practice of Medicine; in which course the diseases of the mind, and those of the body which are more immediately under mental influence, will be especially considered.

A second edition of Mr. Carmichael's "Essay on the Effects of Carbonate, and other Preparations of Iron upon Cancer, with an Enquiry into the Nature of that Disease," is in the press. This edition is so much enlarged and improved, that it may almost be considered a new work. Among the additions are a great number of cases, a disquisition on the uses of oxide of iron in the blood, and remarks on such diseases as depend on its excess or deficiency, or in any way bear a relation to cancer; with an attempt to answer the Queries of the Medical Society, established in London, for investigating the nature and cure of that complaint.

